# Health related quality of life in sepsis survivors from the Prehospital Antibiotics Against Sepsis (PHANTASi) trial

**DOI:** 10.1371/journal.pone.0222450

**Published:** 2019-10-01

**Authors:** R. S. Nannan Panday, T. C. Minderhoud, D. S. Chantalou, N. Alam, P. W. B. Nanayakkara

**Affiliations:** 1 Section Acute Medicine, Department of Internal Medicine, Amsterdam UMC, Vrije Universiteit Amsterdam, Amsterdam, The Netherlands; 2 Amsterdam Cardiovascular Sciences, Amsterdam UMC, University of Amsterdam, Amsterdam, The Netherlands; 3 Amsterdam UMC, Vrije Universiteit Amsterdam, Amsterdam, The Netherlands; University of Pittsburgh, UNITED STATES

## Abstract

**Background:**

Due to the rise in incidence, the long term effect of sepsis are becoming more evident. There is increasing evidence that sepsis may result in an impaired health related quality of life. The aim of this study was to investigate whether health related quality of life is impaired in sepsis survivors and which clinical parameters are associated with the affected health related quality of life.

**Methods:**

We analyzed 880 Short Form 36 (SF-36) questionnaires that were sent to sepsis survivors who participated in the Prehospital Antibiotics Against Sepsis (PHANTASi) trial. These questionnaires were sent by email, 28 days after discharge. Data entry and statistical analyses were performed in SPSS. The data from the general Dutch population, was obtained from the Netherlands Cancer Institute (NKI-AVL) and served as a control group. Subsequently, 567 sepsis survivors were matched to 567 controls. Non-parametric Wilcoxon signed-rank test was performed to compare these two groups. Within the group, we sought to explain the diminished health related quality of life by factor analysis.

**Results:**

We found that sepsis survivors have a worse health related quality of life compared to the general Dutch population. This negative effect was more evident for the physical component than the mental component of health related quality of life. We found that health related quality of life was significantly altered by advancing age and female sex. We also found that the total length of stay (in the hospital) and (previous) comorbidity negatively affect the physical component of health related quality of life.

**Conclusion:**

In our study we found that health related quality of life in sepsis survivors, 28 days after discharge, is severely diminished in comparison with the general Dutch population. The physical domain is severely affected, whereas the mental domain is less influenced. The length of stay, comorbidity, advancing age and female sex all have a negative effect on the Physical Component Scale of the health related quality of life.

## Introduction

Sepsis is a syndrome of physiologic, pathologic and biochemical abnormalities induced by infection [1]. The worldwide incidence of sepsis is rising. This is caused by several factors, such as: the ageing population, antibiotic resistance, increased use of chemo-and immunotherapy and improved recognition [2],[1]. A meta-analysis of 27 international studies reported a global sepsis incidence of 437 per 100,000 person-years for the last decade [3]. Conversely, in-hospital mortality is decreasing. Previous studies found that in-hospital septic shock mortality decreased from 54.9% to 50.7% from 2005 to 2014 [4]. This decrease in case fatality can be attributed to improved recognition, clinical advances including early goal-directed therapy and mortality reduction campaigns [5].

Previous research has shown that HRQoL (Health-Related Quality of Life) is impaired in sepsis survivors [6], [7], [8]. A recent systematic review [2] found that 81.3% of ICU-sepsis survivors reported an impaired quality of life which lasted for years after the syndrome was treated.

However, research conducted on the HRQoL of sepsis survivors has been focused only on ICU patients. No studies have been conducted on the HRQoL in either a population of sepsis survivors with varying severities of sepsis or in sepsis patients who were transported to the ED by EMS.

Studies that compared quality of life of sepsis ICU survivors to the general population in several countries [7–10] found decreased HRQoL of the sepsis ICU survivors when their HRQoL was compared to that of the general population of these countries. However, it is not yet known if this effect is caused by the ICU admission or by the sepsis episode itself.

This prospective study will focus on the quality of sepsis survivors that were transported by EMS to the ED. A study in the Netherlands found that nearly half of all patients presenting to the ED were transported by Emergency Medical Services (EMS), and those transported by EMS were sicker[11]. Therefore this population is a representative sample of severely ill hospitalized sepsis patients.

The patients in this study are those that were included in the Prehospital Antibiotics Against Sepsis (PHANTASi) trial recently published in The Lancet Respiratory Medicine[12, 13].

The primary aim of this study is to evaluate the HRQoL of sepsis survivors. Subsequently the HRQoL of sepsis survivors was compared to the general Dutch population in order to determine to what extent the HRQoL of sepsis survivors differs from the general Dutch population. The secondary aims were to analyze which patient characteristics and clinical parameters were associated with the decreased HRQoL in sepsis survivors.

## Methods

### Design and setting

This prospective study was part of the PHANTASi trial[12, 13]. In brief, the PHANTASi trial was the first prospective randomized controlled trial in septic patients which investigated whether improved recognition and administration of antibiotics in the ambulance led to increased survival when compared to usual care. Sepsis severity was categorized in to three groups as defined by the 2001 SSCM/ ESCIM/ ACCP/ ATS/ SIS International Sepsis definitions Conference guidelines: (uncomplicated) sepsis, severe sepsis and septic shock. [14]. The sepsis diagnosis was cross-checked at the ED by the attending physician. Patients characteristics and clinical parameters such as Charlson Comorbidity Index, clinical values, laboratory values, sepsis severity, organ dysfunction, hospital and ICU length of stay and readmission among others, were derived from patients medical charts.

### Methodology

All patients in the study that survived their hospital admission were sent a SF-36 questionnaire [15] by mail within one month after discharge in order to measure their HRQoL. The SF-36 is a widely used [16], standardized questionnaire for measuring HRQoL. This questionnaire has been validated for the measurement of HRQoL in sepsis survivors [9] and is also validated in the Dutch Language [17]. The SF-36 measures HRQoL by addressing eight domains: physical functioning, role limitation due to physical problems, role limitation due to emotional problems, social functioning, bodily pain, mental health, vitality and general health. These eight domains are clustered into two summary scores: a physical component score (PCS) and a mental component score (MCS) [18]. The PCS consists of the domains: Physical Functioning, Role Functioning Physical, Bodily Pain and General Health. The MCS consist of the domains: Vitality, Social Functioning, Role Functioning Emotional and Mental Health.

### Outcomes

The primary outcome is the HRQoL in sepsis survivors in this Dutch population compared to the HRQoL of the General Dutch population. To increase our understanding of the potential changes in HRQoL we we examined which clinical parameters were associated with the HRQoL. For this, we studied parameters that were associated with HRQOL in previous studies (17–19). These factors consisted of demographic characteristics (age/sex), overall comorbidity (charlson comorbidity index), and specific comorbidity such as chronic pulmonary disease, heart failure, diabetes and cancer), sepsis severity, organ dysfunction and length of hospital stay.

### Statistical analysis

Data are expressed as means and standard deviation (SD ±) if the data is normally distributed or median and interquartile range (IQR) if the data exhibited a non-normal distribution.

Raw scores from each of the 36 items were entered into SPSS (IBM version 22.0). These raw scores were transformed to scores that ranged from 0 to 100 as per the guidelines of the RAND corporation [19]. Higher scores of any domain correspond with a better HRQoL. Differences between SF-36 domains per age category were analysed by non-parametric Kruskal-Wallis one-way-ANOVA. A p-value < 0.05 was considered statistically significant.

Matching In order to compare the SF-36 data of our sepsis survivors population to the general Dutch population we retrieved data from the Netherlands Cancer Institute (NKI-AVL) [17]. This general Dutch population did not necessarily contain patients with malignant diseases, as it was a representative sample of the general Dutch population, which was used in a previous study. The data in that study was retrieved by sending out questionnaires to a randomly selected population in the Netherlands[17]. The age and gender matched data was compared by using a non-parametric Wilcoxon signed-rank test. Patients were matched by gender with a maximum age difference of 5 years between those in the study group and those in the control group. No information on the comorbidities of the patients in the control group was known and therefore matching for this aspect was not possible.

To analyse clinical parameters associated with HRQoL, non-parametric Mann-Whitney U test and Kruskal-wallis-1-way-ANOVA were used for prespecified factors. Clinical parameters with a P < 0.05 were included in a multiple linear regression analysis by blockwise entry.

### Ethics

The study protocol of the PHANTASi trial was approved by the medical ethical committee of the Amsterdam University Medical Center, Location VU University Medical Center, the coordinating center and all ethical bodies of each participating hospital. Due to the complexity of the PHANTASi trial, the ethics committees granted approval to obtain deferred consent when necessary. Informed consent before study enrollment or deferred consent was obtained from all patients or their legal representatives or surrogates. All effort was made by EMS personnel to obtain informed consent before study inclusion provided the acuity of the situation allowed it.

## Results

### Patient characteristics and demographics

**Fig 1:**
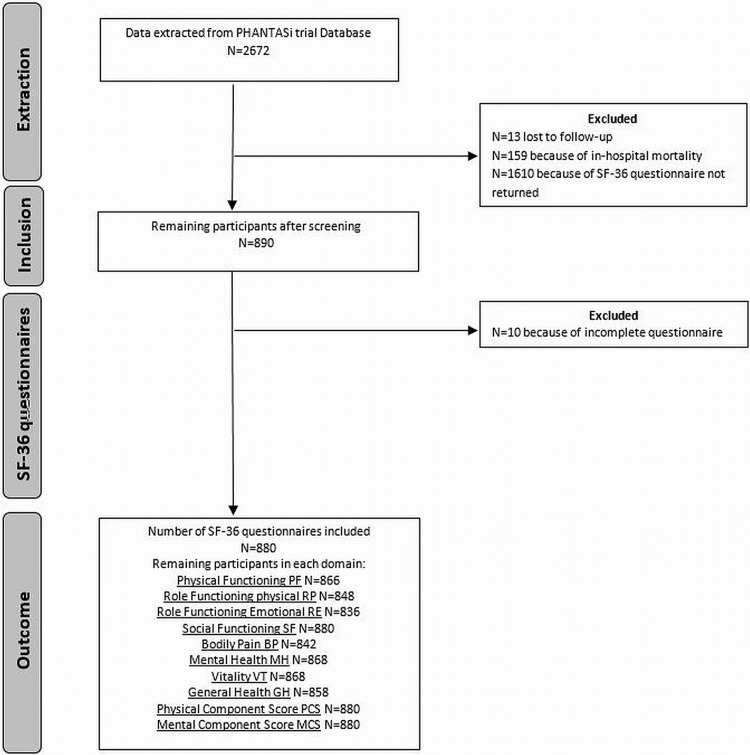
Overview of patients included in the study.

The PHANTASi included patients who had sepsis according to the SEPSIS-2 criteria which were used in the study. The SEPSIS-2 criteria are more sensitive but less specific in diagnosing sepsis[20]. 2672 patients were included in the PHANTASi trial. 13 patients were lost to follow-up and 159 patients did not survive their hospital admission. 1610 patients did not return the questionnaire or were excluded due to incomplete questionnaire. A total of 880 questionnaires remained for analysis (Fig 1).

The 880 patients who returned the questionnaire had a mean age of 72.8 ± 12.6 and 58.9% were male (Table 1). There were a number of important differences between patients who did and did not return the questionnaire. The patients who did not return the questionnaire had a significantly higher Charlson Comorbidity Index (p = .016), and had significantly more cerebrovascular diseases and dementia (p < .001). Patients who did not return the questionnaire had a higher 90 day mortality rate of 5.2% versus 1.0% (p < .001).

**Table 1:** Demographic and characteristics of the PHANTASi trial patients (Total N = 2649).

Characteristics	SF-36 questionnaire returned (N = 880)	SF-36 questionnaire not returned (in hospital mortality excluded)(N = 1610)	*P*
Age-years	72.9 ±12.51	72.0 ± 12.51	.132^
Male sex–no (%)	516 (58.9)	869 (56.4)	.230*
Charlson Comorbidity Index (median/IQR)	1 (0–2)	1 (1–3)	.016 ^#^
**Underlying chronic conditions (%)**			
Chronic pulmonary disease	262 (29.9)	460 (29.9)	.976*
Diabetes	205 (23.4)	372 (24.1)	.682*
Malignancy <5yrs	113 (12.9)	198 (12.8)	.971*
Congestive heart failure	75 (8.6)	166 (10.8)	.081*
Dementia	19 (2.2)	95 (6.2)	< .001*
**Severity of sepsis (%)**			
Sepsis	360 (41.6)	608 (40.2)	.508*
Severe sepsis	477 (55.1)	863 (57.0)	.354*
Septic shock	26 (3.0)	41 (2.7)	.678*
**Admission (%)**			
Hospital	849 (96.9)	1458(94.6)	.009
ICU	78 8.9%)	123(8.0)	.77*
Readmission	56 (6.4%)	125 (8.1%)	.123
**Length of stay (median/IQR)**			
Hospital	5 (4–8)	6(4–10)	.961^#^
ICU/ MCU	0 (0–0)	0 (0–0)	.296^#^
**Mortality (%)**			
90 days	8 (1.0)	80 (5.2)	< .001*

Data are presented as N (%), mean (SD/±) or median (IQR = Inter Quartile Range). ED = Emergency Department. ICU = Intensive Care Unit.

^independent samples T-test

* Chi square test

^#^ Mann whitney U test

Data are presented as N (%), mean (SD/±) or median (IQR = Inter Quartile Range). ED = Emergency Department. ICU = Intensive Care Unit.

### Primary outcome

#### Sepsis survivors compared to the general Dutch population

Sepsis survivors, 4 weeks after discharge, had a statistically significant lower median Physical Component Score of 33.2 (IQR 26–43) and Mental Component Score of 45.4 (IQR35-53) compared to the matched general Dutch population which had a median 48.3 (IQR 38–54) and median 54 (IQR 47–58) respectively. This difference was significant for both PCS and MCS (p< 0.001).

#### SF-36 HRQoL subdomains

The different subdomains also exhibit a statistically significant lower SF-36 score compared to the General Dutch population (Table 2).

**Table 2:** SF-36 scores of Sepsis Survivors compared to general Dutch population.

	Median SF-36 scores		
SF-36 Domains	Sepsis Survivors N = 567	Matched General Dutch Population N = 567	*P*
**Physical functioning (PF)N = 553**	38.9 (15–75)	80 (55–90)	< .001
**Role functioning Physical (RF) N = 520**	0 (0–50)	100 (25–100)	< .001
**Role functioning Emotional (RE) N = 509**	66.7 (0–100)	100 (66.7–100)	< .001
**Social functioning N = 563**	62.5(38–75)	87.5 (63–100)	< .001
**Bodily Pain N = 543**	63.3 (IQR 35–90)	74 (IQR 51–100)	< .001
**Mental Health N = 546**	72 (IQR 52–84)	80 (IQR 64–88)	< .001
**Vitality N = 546**	45 (IQR 30–60)	70 (IQR 55–80)	< .001
**General Health N = 532**	40 (IQR 20–55)	67 (IQR 50–77)	< .001
**Physical component score N = 514**	33.2 (26–43)	48.3 (IQR 38–54)	< .001
**Mental component score N = 514**	45.4 (IQR 35–53)	54(IQR 47–58)	< .001

### Health related quality of life in sepsis survivors

**Fig 2:**
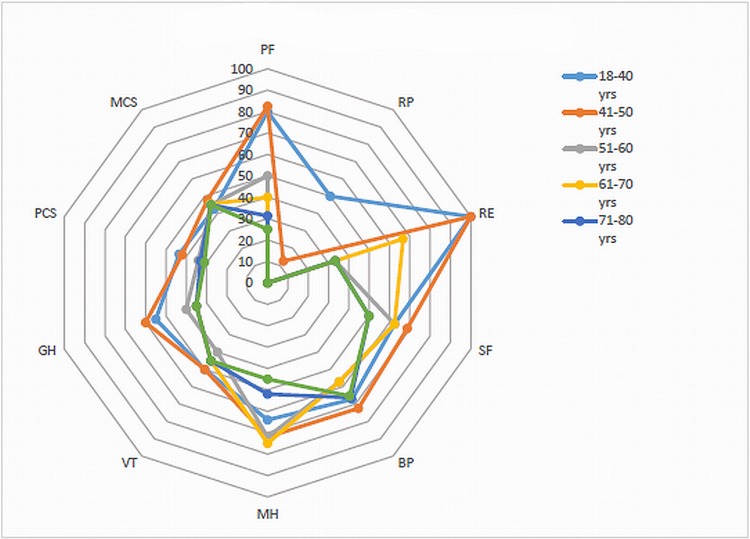
Spyder diagram, HR-QOL by age category.

Sepsis survivors had a median Physical Component Score (PCS) of 32.9 (IQR 26–41) and a median Mental Component Score (MCS) of 45.1 (IQR 35–53) (Table 2). When PCS is divided per age category, older respondents have significant worse scores compared to younger respondents (p<0.001). MCS did not differ throughout the different age groups (p = 0.970). Sepsis survivors scored lowest for the Role Functioning Physical domain (median: 0.0, IQR 0–50) and the highest for the Mental Health domain (median = 72, IQR = 55–84). Physical Functioning, Role Functioning Physical, Role Functioning Emotional and General Health exhibit an overall decline with increasing age: (p < 0.001, p = 0.025, p < 0.001 and p = 0.022 respectively) (Fig 2).

### Secondary outcome: What are contributing factors for decreased HRQoL

#### Age & sex

Bivariate analysis showed a significant, negative effect of age on the PCS (p = .002). The MCS however was not significantly affected by age (p = .765). Female sex was also associated with a small but significantly worse score on the PCS (p = .024), but likewise not in the MCS.

#### Length of stay and ICU versus hospital admission

ICU admission was not significantly associated with MCS or PCS scores (p = .063 for the PCS and p = 0.349 for the MCS). The total length of stay (including ICU days) was significantly associated with both MCS and PCS: an increase in length of stay resulted in a significant decrease in the PCS and MCS with p < .001.

### Comorbidities and the effect on quality of life

A Charlson Comorbidity Index (CCI) > 3 was significantly associated with a lower PCS score (p < .001), patients with a CCI 0–3 had a median score of 33.4 (IQR 26–43) compared to a median score of 30.2 (IQR 23–35) in patients with a CCI > 3. MCS was also significantly associated with CCI (p = .039). Patients with CCI > 3 had a median score of 41.9(IQR 32-51-51) compared to patients with a CCI score of 0–3 with a median score of 45.7 (IQR 35–54). Chronic (Obstructive) pulmonary disease, heart failure and diabetes all had a significant negative effect on the PCS compared to patients without comorbidity. The effect on MCS was not significant, except for patients with diabetes, who had a significantly worse median MCS of 41.7 versus 45.7 in patients without diabetes. Strikingly, malignancy (in the past 5 years) showed no significant effect on either PCS and MCS in the mann-whitney u test (p = .18 and p = .52 respectively).

### Organ dysfunction and sepsis severity

Patients with Central Nervous System (CNS) organ dysfunction had a median MCS of 42.6 (IQR 31–52) versus 45.6 (IQR 36–54). CNS organ dysfunction was negatively associated with a lower MCS (p < .001). Other differences in organ dysfunction such as renal or pulmonary dysfunction did not show significant effects on either the PCS or MCS. Sepsis severity studied by performing a Mann Whitney U test comparing patients with sepsis alone (SEP 2 definition) to the more severe forms (severe sepsis and septic shock). For the PCS, the group with sepsis alone had a median PCS of 34.0, whereas patients with more severe forms of sepsis had a median PCS of 31.8. This was a significant difference with a p-value of .044.

The median MCS is in the group with sepsis was 44.3, compared to 45.6 in the group with more severe sepsis, which was not significantly different (p-value = .65)

### Regression model

As stated before, all independent factors with a significant association were then studied in a linear regression model. For the PCS the following parameters were entered in the multiple regression model: age, sex, heart failure, diabetes, chronic pulmonary disease, sepsis severity and total length of stay. In the regression model, sepsis severity did not show a significant association with PCS with a p-value of .79 and was therefore excluded in the final model. Diabetes also did not show a significant association with a p-value of .058 and was excluded. The final model is displayed in Table 3. Age is negatively associated with PCS. For every year increase in age, the PCS decreased -0,14 (95%CI -.20 to– 0.08). Female sex showed a negative association with PCS, with a decrease of 1.93 (95%CI -3.42 to– 0.44) point on the PCS for females compared to males. A history of heart failure resulted in a decrease of 3.99 (95%CI -6.62 to -1.37) points on the PCS. Chronic pulmonary disease had the greatest effect on the PCS, with a decrease of 4,38 (95%CI -5.99 to -2.77).For MCS, sex, length of stay, diabetes and CNS organ dysfunction were entered in the model. Sex did not show significant association in the regression model and was excluded in the final model. The final model is displayed in Table 4. The presence of CNS organ dysfunction during the admission was associated with a decrease of the MCS of 2,56 points (95%CI -4.68 to -.45). Having diabetes also decreased MCS, with 2.59 points (95%CI -4.46 to -.73) Length of stay was also negatively correlated, for every day in the hospital the MCS decreased with .28 points (95%CI -4.46 to -.73)

**Table 3:** Linear regression: Factors associated with the physical component score.

		Unstandardized regression coefficient	95% Confidence interval	Standardized regression coefficient	P-value
**Final model^a^**	**Age**	-0.14	-.20 to -.08	-.15	.000
	**Sex**	-1.93	-3.42 to -.44	-.09	.011
	**Heart failure**	-3.99	-6.62 to -1.37	-.10	.003
	**Chronic Pulmonary disease**	-4.38	-5.99 to -2.77	-.18	<001
	**Length of stay (total)**	-0.23	-.33 to -.12	-.14	< .001
			-2.38		
			-4.90		
			-2.73		

^a^Variables entered in the equation stepwise were age, sex, heart failure, diabetes, chronic pulmonary disease, sepsis severity and total length of stay. Variables with a p-value < .05 were included in the final model.

**Table 4:** Linear regression: Factors associated with the mental component score.

		Unstandardized regression coefficient	95% Confidence interval	Standardized regression coefficient	P-value
**Final Model^a^**	CNS organ dysfunction	-2.56	-4.68 to -.45	-.08	.018
	**Length of stay (total)**	-.28	-,40 to -,17	-.17	<,001
	**Diabetes**	-2.59	-4.46 to -,73	-.09	.006

^a^Variables entered in the equation were sex, length of stay, diabetes and CNS organ dysfunction. Variables with a p-value < 0,05 were included in the final model.

## Discussion

In this study on Health Related Quality of Life in sepsis survivors we investigated HRQoL by analyzing SF-36 questionnaires returned 28 days after discharge by sepsis survivors who were transported by ambulance to the ED. We found that sepsis survivors, 28 days after discharge had a significantly worse HRQoL compared to the Dutch general population. This negative effect was more evident for the physical partof the HRQoL compared to the mental part. In line with previously published data[21], we found a decrease of the physical component score (PCS) with increasing age. Regarding the clinical and vital parameters associated with HRQoL in sepsis survivors, our findings showed that the total length of stay (hospital and ICU length of stay combined), comorbidity such as heart failure and chronic pulmonary disease have a significant negative association with the physical component of the HRQoL.

The mental component score in our cohort was significantly associated with length of stay, but not age or sex. Of other factors described in literature, only diabetes and CNS organ dysfunction showed a significant, negative association with the mental component of HRQoL.

Our findings regarding the decreased HRQoL in our cohort of sepsis survivors, is similar to those of studies conducted in ICU sepsis survivors [2, 6–10]. Our results indicate that this negative effect tends to be greater for the domains regarding the physical component compared to the domains regarding the mental component.

Previous studies which compared quality of life of ICU sepsis survivors to the General French population [10], Scottish population [8], U.S. population [9] and Dutch population [7], show similar results. The fact that we found similar results in our study population, suggests that these effects can not only be attributed to the ICU admission, since only a small proportion of our study population was admitted to the ICU.

These studies also consistently show that not all of the domains are always negatively affected. Heyland and colleagues [9] compared the quality of life of 30 ICU sepsis survivors to the general US population, at discharge and two weeks after discharge. They found that the components related to physical and social function were lower than the US population, but Bodily Pain, Role Emotional, Mental Health and Mental Component score were not significantly lower. Another explanation why the MCS seems less affected by sepsis, may lie in the so called response shift. Response shift is defined as a change in a person’s perception of his own quality of life due to recalibration, reprioritization or redefinition of a person’s value of a ‘good’ quality of life which can occur after an experience of hardship [22]. Thus this may explain why subjective measures seem to be minimally affected by sepsis. This might also explain the effect that we found septic shock survivors to be 2.5 times more likely to have a good Role Functional Emotional score compared to (uncomplicated) sepsis survivors. Granja and colleagues [23] assessed HRQoL by using the EuroQol five dimensions (EQ-5D) questionnaire and found that sepsis survivors (septic shock and severe sepsis combined) have fewer problems on the depression and anxiety dimension compared to survivors of other critical illness.

### Factors influencing PCS

For our secondary outcomes we observed that hospital length of stay and comorbidity such as heart failure, chronic pulmonary disease negatively affect the Physical Component Score. An association between pulmonary dysfunction and decreased quality of life are provided by several studies (10). In regards to the negative association of hospital stay and HRQoL, length of stay has been described to be a risk factor for impaired Physical Functioning [24]. Furthermore, there is evidence that bed rest may lead to muscle wasting [25].

Female sepsis survivors were found to have a lower physical component score of the HRQoL in our study, even after correcting for other factor such as age. We found no explanation for this difference, however studies on other subjects also found women to have lower general HRQoL than men, without an explanation for this phenomenon. Although some studies suggest that this difference might be caused by sociodemographic differences, no definitive explanation for this effect is known[26–29].

### Factors influencing MCS

We found that central nervous system dysfunction and diabetes t both have a negative effect on the Mental Component score. Previous literature about the association between delirium an HRQoL are not consistent. Prior mental status has a negative effect on impaired quality of life as seen in the study conducted by Davydow and colleagues [30]. However, Boogard and colleagues [31] suggest that other factors associated with delirium instead of delirium itself may explain overall lower SF-36 scores as they found that patients with delirium exhibit no significant difference in HRQoL compared to patients without delirium. We found that diabetes mellitus had a negative effect on the Mental Component Score. There is evidence that the negative effect of diabetes on HRQoL is not due to the duration and type of diabetes itself, but that secondary complications, demographical and psychosocial factors have a negative effect [32] [33] [34].

Our findings regarding the impaired Health Related Quality of Life after sepsis and the associated clinical parameters, give us leads for possible interventions to ensure a better HRQoL. Interventions are important since HRQoL has a big impact on patients life for example in personal relationships [30] or might also be a financial burden [35]. One study protocol regarding a double-blinded randomized controlled trial analyzing the effect of a multidisciplinary intervention in sepsis survivors has been published. Data has yet to be published [36].

### Strengths and weaknesses

Our study contains several strengths. Firstly, the overall number of returned SF-36 questionnaires are relatively high compared to other studies [7, 8, 10, 37]. To the best of our knowledge we have the largest sample of sepsis survivors who were timely recognized and treated by trained medical personnel. Moreover we are also the first to study HRQoL of sepsis survivors transported to the ED by ambulance, thus not restricted only to patients admitted to the ICU. Several other studies have compared quality of life to the general population and to our knowledge only one study used the adult general Dutch population [7]. What differentiates our study compared to Hofhuis and colleagues is that we used a large sample of the norm population which we matched by age and sex. Additionally, the patients included in this study come from different cities from the Netherlands including both urban and rural areas. Therefore a possible bias in quality of life due to a urban or rural setting was avoided [38]. Lastly, the SF-36 questionnaires were all self-administered in contrast to some studies which administered the questionnaire via telephone. Patients in phone interviews have a tendency to give socially desirable answers in contrast to patients who fill out the questionnaires themselves, possibly due to perceived anonymity [39, 40]. Thus self-administered questionnaires may portray more accurate answers.

Our study also holds several limitations. Firstly, we did not have a baseline measure of HRQoL, thus firm conclusions regarding impaired HRQoL solely influenced by sepsis cannot be made as several factors such as patient characteristics and premorbid quality of life may affect HRQoL [41]. Although the data of sepsis survivors was matched for age and sex, we were unable to match for comorbidities in the general Dutch population as this information was not available. However, previous studies have consistently shown that survivors of other acute illnesses also have a lower HRQoL when compared to the general population[42, 43].

Second, another limitation of our study is the lack of follow-up. Measuring the HRQoL one month after discharge gives the patient little time to recuperate. We chose this short time span, contrary to other HRQoL studies as other sepsis studies focused exclusively on ICU patients[2]. In our study population, 9,4% of patients were admitted to the ICU[12]. Therefore our study population was less severely ill which made us choose shorter follow-up period as they might require less time to recover. This is supported by the median duration of hospitalization of 5 days. Our study shows that even in patients not admitted to the ICU, the effect on PCS is still large, albeit relatively short after admission.

Third, at the time of inclusion of patients in this study, the SEPSIS-2 criteria were still the gold standard, which have nowadays been replaced by the SEPSIS-3 criteria, which would mean that our sample size would have been less if patients were included according to the new criteria as these are less sensitive than the SEPSIS-2 criteria[20]. However, the fact that patients who were included by using the more sensitive SEPSIS-2 criteria still suffer from decreased HRQoL, underlines the fact that this diminished HRQoL might be even worse if patients were to be included by using the SEPSIS-3 criteria.

Our sample was not completely representative of all patients in our study, as patients that returned the questionnaire had a significantly lower CCI compared to patients that did not return the questionnaire. However, earlier studies (8–10) have already focused on the group of patients most severely ill (those in ICU), and show similar conclusions.

## Conclusion

In a cohort of sepsis-survivors, 28 days after discharge, we found that HRQoL is considerably lower than the general Dutch population. This effect is most profound on the Physical Component Score. Length of stay and comorbidity, especially heart failure and chronic pulmonary disease, are significantly associated with a lower HRQoL in the physical domain. HRQoL.

## Supporting information

S1 Appendix SF-36 scores in sepsis survivors by age category. PF = Physical Functioning, RP = Role Functioning Physical, BP = Bodily Pain, GH = General Health, VT = Vitality, SF = Social Functioning, RE = Role Functioning Emotional, MH = Mental Health, PCS = Physical Component Score and MCS = Mental Component Score. * See appendix for supplementary figures for number of patients included in each age group.
(DOCX)
