# Supplementary material for: Health related quality of life in sepsis survivors from the Prehospital Antibiotics Against Sepsis (PHANTASi) trial
Source: PLoS One. 2019 Oct 1;14(10):e0222450. doi: 10.1371/journal.pone.0222450 (PMC6772145; doi:10.1371/journal.pone.0222450)
Supplement: S1 Appendix — PF = Physical Functioning, RP = Role Functioning Physical, BP = Bodily Pain, GH = General Health, VT = Vitality, SF = Social Functioning, RE = Role Functioning Emotional, MH = Mental Health, PCS = Physical Component Score and MCS = Mental Component Score. * See appendix for supplementary figures for number of patients included in each age group. (DOCX) [file pone.0222450.s001.docx]

|  | | **SF-36 scores of Sepsis Survivors** | **SF-36 scores of sepsis survivors by age group** | | | | | | |
| --- | --- | --- | --- | --- | --- | --- | --- | --- | --- |
| **SF-36**  **Domains** | **N=880** | **18-40 N=23*** | **41-50 N=23-24** | **51-60 N=75-77** | **61-70 N=184-192** | **71-80**  **N=292-307** | **>81 N=239-257** | ***P*** |  |
| **PF**  **N=866** | 35  (10-65) | 80 (55-95) | 82.5  (50-98) | 50 (20-79) | 40 (15-75) | 31.3 (10-65) | 25 (5-50) | <0.001 |  |
|  | **RP**  **N=848** | 0  (0-50) | 50 (0-100) | 12.5 (0-100) | 0  (0-25) | 0  (0-50) | 0  (0-37.5) | 0  (0-50) | 0.025 |
| **RE**  **N=836** | 33.3  (0-100) | 100  (33-100) | 100  (100-100) | 33.3  (0-100) | 66.7  (0-100) | 33.3  (0-100) | 33.3  (0-100) | 0.002 |  |
| **SF**  **N=880** | 62.5  (38-75) | 62.5  (38-81) | 68.8  (44-94) | 62.5  (25-75) | 62.5  (38-75) | 50  (38-75) | 50  (38-75) | 0.588 |  |
| **BP**  **N=842** | 65.3  (35-88) | 67.3  (33-90) | 72.4  (55-100) | 57.1  (39-78) | 57.1  (38-90) | 66.3  (33-90) | 65.3  (35-80) | 0.776 |  |
| **MH**  **N=868** | 72  (55-84) | 64  (60-90) | 72  (58-82) | 72  (56-80) | 75  (52-84) | 52  (52-84) | 45  (30-60) | 0.948 |  |
| **VT**  **N=868** | 45  (30-60) | 50  (30-65) | 50  (30-73) | 40  (26-58) | 45  (30-60) | 45  (30-65) | 45  (30-60) | 0.676 |  |
| **GH**  **N=858** | 35  (20-55) | 55  (40-75) | 60  (18-65) | 40  (20-55) | 35  (20-60) | 35  (25-50) | 35  (25-50) | 0.022 |  |
| **PCS**  **N=880** | 32.9  (26-41) | 43.5  (35-53) | 42.1  (33-52) | 34  (28-42) | 32.2  (25-45) | 33.0  (25-41) | 31  (26-39) | <0.001 |  |
| **MCS**  **N=880** | 45.1  (35-53) | 42.6  (33-56) | 47.9  (38-54) | 45  (33-53) | 45.5  (34-53) | 45  (35-53) | 45.1  (35-54) | 0.970 |  |

Data are presented as median and Inter Quartile Range (IQR).
